# Thyroid stimulating hormone stimulates the expression of glucose transporter 2 via its receptor in pancreatic *β* cell line, INS-1 cells

**DOI:** 10.1038/s41598-018-20449-3

**Published:** 2018-01-31

**Authors:** Jingya Lyu, Hitomi Imachi, Takuo Yoshimoto, Kensaku Fukunaga, Seisuke Sato, Tomohiro Ibata, Toshihiro Kobayashi, Tao Dong, Kazuko Yonezaki, Nao Yamaji, Fumi Kikuchi, Hisakazu Iwama, Ryou Ishikawa, Reiji Haba, Yasunori Sugiyama, Huanxiang Zhang, Koji Murao

**Affiliations:** 1 Department of Endocrinology and Metabolism, Faculty of Medicine, Kagawa University, 1750-1, Miki-cho, Kita-gun, Kagawa, 761-0793 Japan; 2 Department of Cell Biology, Medical College of Soochow University, Jiangsu Key Laboratory of Stem Cell Research, Ren Ai Road 199, Suzhou Industrial Park, Suzhou, 215123 China; 3 Life Science Research Center, Kagawa University, 1750-1, Miki-cho, Kita-gun, Kagawa, 761-0793 Japan; 4 Department of Diagnostic Pathology, Kagawa University Hospital, 1750-1, Miki-cho, Kita-gun, Kagawa, 761-0793 Japan; 5 Department of Life Sciences, Faculty of Agriculture, Kagawa University, 2393, Miki-cho, Kita-gun, Kagawa, 761-0795 Japan

## Abstract

Thyroid stimulating hormone (TSH) stimulates the secretion of thyroid hormones by binding the TSH receptor (TSHR). TSHR is well-known to be expressed in thyroid tissue, excepting it, TSHR has also been expressed in many other tissues. In this study, we have examined the expression of TSHR in rat pancreatic islets and evaluated the role of TSH in regulating pancreas-specific gene expression. TSHR was confirmed to be expressed in rodent pancreatic islets and its cell line, INS-1 cells. TSH directly affected the glucose uptake in INS cells by up-regulating the expression of GLUT2, and furthermore this process was blocked by SB203580, the specific inhibitor of the p38 MAPK signaling pathway. Similarly, TSH stimulated GLUT2 promoter activity, while both a dominant-negative p38MAPK α isoform (p38MAPK α-DN) and the specific inhibitor for p38MAPK α abolished the stimulatory effect of TSH on GLUT2 promoter activity. Finally, INS-1 cells treated with TSH showed increased protein level of glucokinase and enhanced glucose-stimulated insulin secretion. Together, these results confirm that TSHR is expressed in INS-1 cells and rat pancreatic islets, and suggest that activation of the p38MAPK α might be required for TSH-induced GLUT2 gene transcription in pancreatic *β* cells.

## Introduction

Thyroid stimulating hormone (TSH), also known as thyrotropin, belongs to a pituitary glycoprotein hormone family. Secretion of TSH from the pituitary is stimulated by thyrotropin-releasing hormone (TRH) from the hypothalamus. Once secreted, TSH mainly acts to stimulate the thyroid by binding its receptor, TSH receptor (TSHR)^1^. TSHR is a member of the G protein-coupled receptor family and is an 82-kDa protein composed of α and β subunits^2^. Activation of TSHR leads to the transcription, synthesis and release of thyroid hormones via the PKA signaling pathway within the thyroid. Excepting thyroid tissue, TSHR has also been reported to be expressed in many other tissues and cells, such as the brain, testes, kidney, heart, bone, adipose tissues, thymus, lymphocytes and fibroblasts^2,3^. These varying locations of TSHR expression indicate its capacity to perform multifunctional roles throughout the body, in addition to its best-known role in the thyroid. Recently, TSHR is reported to be expressed in rabbit pancreatic islets and it suggests that TSH may directly mediate the growth of pancreatic islets by TSHR^4^.

In clinical, the glucose-stimulated insulin secretion (GSIS) is elevated in the patient with Grave’s disease (GD, hyperthyroidism), in which the anti-TSHR antibody activates TSHR without TSH^5,6^. In the other hand, high level of TSH in Hashimoto’s disease (hypothyroidism) also increased serum insulin concentration^7^, suggesting that activation of TSHR may affect insulin secretion. Glucose transporter 2 (GLUT2), which is present within the plasma membrane of pancreatic *β* cells^8^, plays an important role in glucose-induced insulin secretion from pancreatic *β* cells by catalyzing the uptake of glucose into the cell^9^. It is a facilitative glucose transporter, and its expression is strongly reduced in glucose-unresponsive islets in various animal models of diabetes^9,10^. GLUT2 contributes to the sensing of glucose not only by fueling the metabolic signaling cascade, but also by triggering a specific protein kinase A signaling pathway^11^. Indeed, GLUT2 cannot always be replaced by alternative GLUT isoforms, suggesting that it has unique qualities^12^. Studies using *β* cells that are engineered with various GLUT isoforms to provide a similar glucose flux showed that only GLUT2 facilitates normal insulin production in response to glucose sensing^13^.

Clinical study showed a relationship between a low level of thyroid hormones and diabetes^14^. In addition, serum TSH has been reported to be positively related to insulin concentration^15^. However, little is known about the direct effect of TSH and TSHR on pancreatic specific genes. In this study, we evaluated the role of TSHR in regulating the expression of pancreas specific-genes including GLUT2 by the stimulation of TSH.

## Results

### Characterization of TSHR expression in the rat pancreatic *β* cells

**Figure 1:**
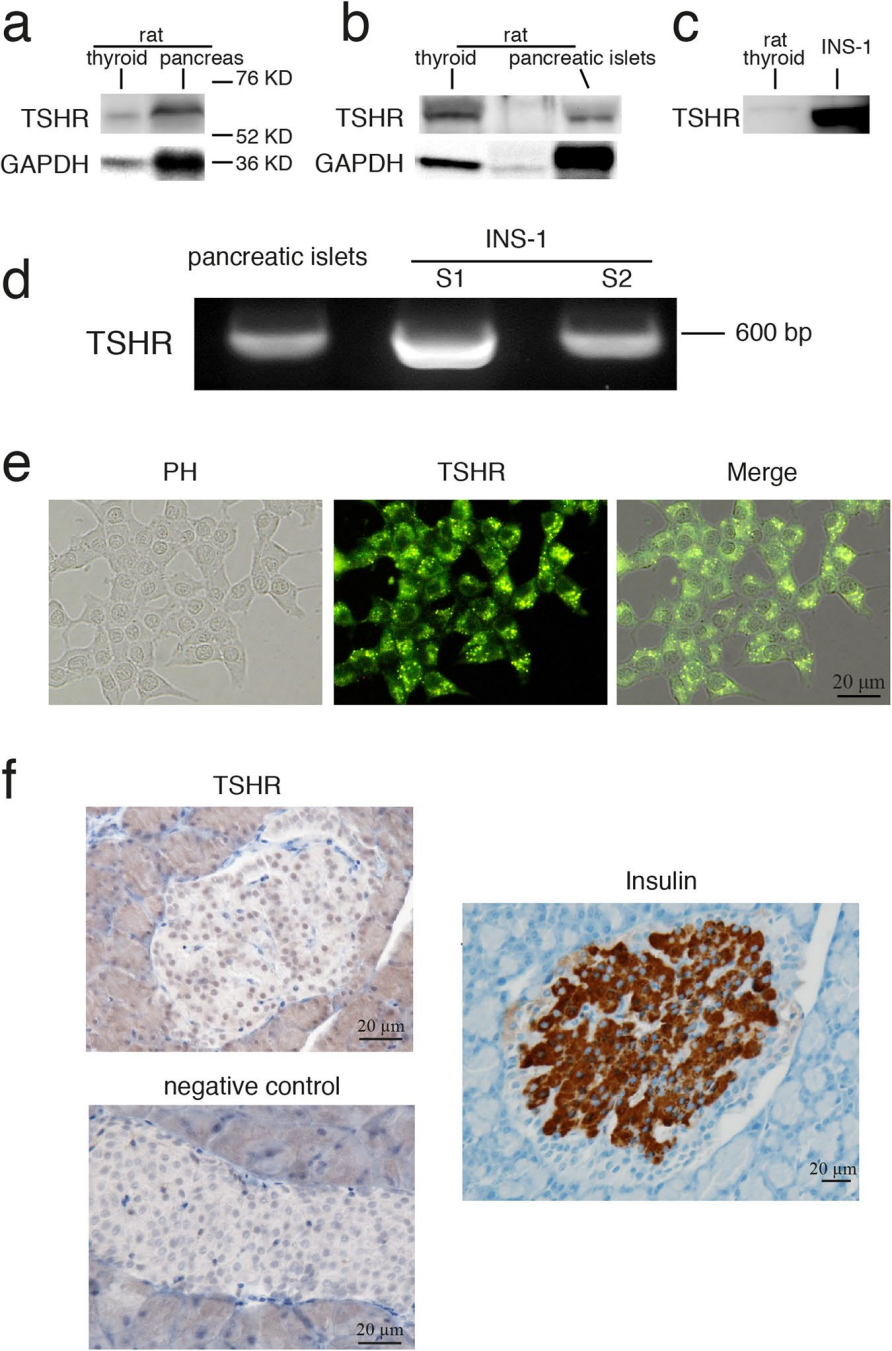
Characterization of TSHR expression in rat pancreatic islets and in INS-1 cells. (**a–c**) Protein isolated from rat thyroid, pancreas, pancreatic islet or INS-1 cells (shown on the top of each band) was detected by TSHR α antibody. Full-length blots are presented in Supplementary Figure 1. (**d)** By using TSHR specific primer, PCR products were generated based on template cDNA isolated from rat pancreatic islets and two dishes of INS-1. (**e**) Immunocytochemistry of INS-1 cells with the TSHR α subunit antibody (green). (**f**) Immunohistochemistry of TSHR and insulin (brown) in primary pancreatic islets from the rat. Bar = 20 µm.

To confirm that TSHR is expressed in the rat pancreas, we used an antibody against the TSHR α subunit and detected a 62-kDa band in the rat pancreas, INS-1 cells, pancreatic islets isolated from rat and the rat thyroid (positive control) (Fig. 1a–c). Using the same primers that were reported to successfully amplify the fragment of TSHR in rats^16^, we generated a 594-bp PCR product from the template cDNA isolated from rat pancreatic islets and INS-1 cells (Fig. 1d). Finally, immunocytochemistry and immunohistochemistry demonstrated that TSHR is expressed in both INS-1 cells (Fig. 1e) and in rat pancreatic islets (Fig. 1f).

### TSH increases the expression of GLUT2 via p38MAPK in rat pancreatic *β* cells

**Figure 2:**
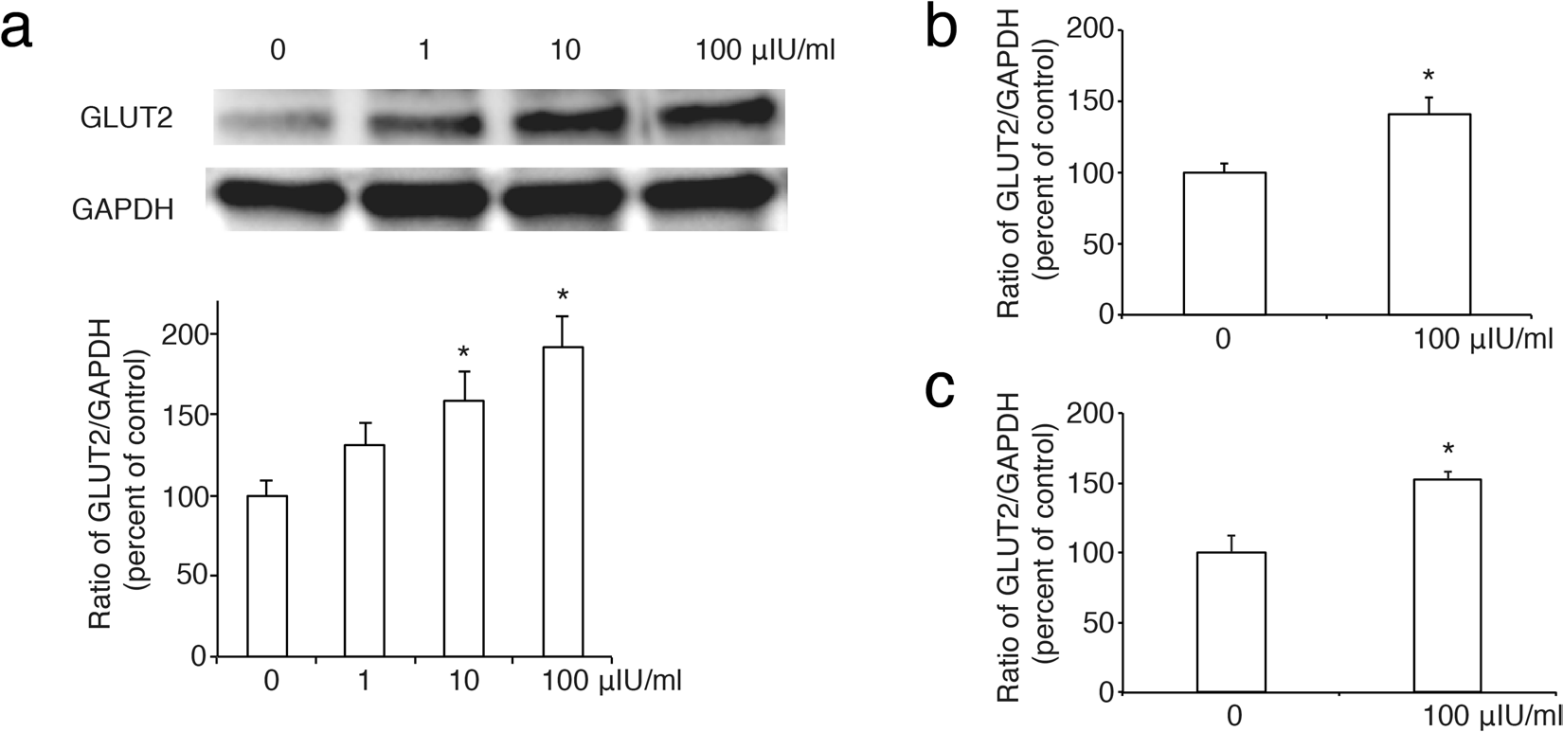
Roles of TSH in the expression of GLUT2 in pancreatic *β* cells. (**a**), TSH increases the abundance of GLUT2 protein in a dose-dependent manner. The ratio of GLUT2 to GAPDH is shown as a percentage of the control ratio. Full-length blots are presented in Supplementary Figure 2. (**b**) and (**c**), The effect of TSH at 100 µIU/ml on GLUT2 mRNA expression by real-time PCR in INS-1 cells (**b**) and pancreatic islets (**c**). GAPDH is used as a control. A graph showing the mean ± SEM (n = 3) of separate experiments for each treatment group is indicated. The *denotes a significant difference (P < 0.05) compared to 0.

Having confirmed that TSHR is expressed in rat pancreatic islets and in INS-1 cells, we then used INS-1 cells to analyse the effects of TSH on the expression of pancreas-specific gene in pancreatic *β* cells. Firstly, we found that TSH increased the expression of GLUT2 in a dose-dependent manner, as indicated by Western blot analysis (Fig. 2a) and real-time PCR (Fig. 2b), respectively. At the same time, this result was confirmed in pancreatic islets isolated from rat (Fig. 2c).

**Figure 3:**
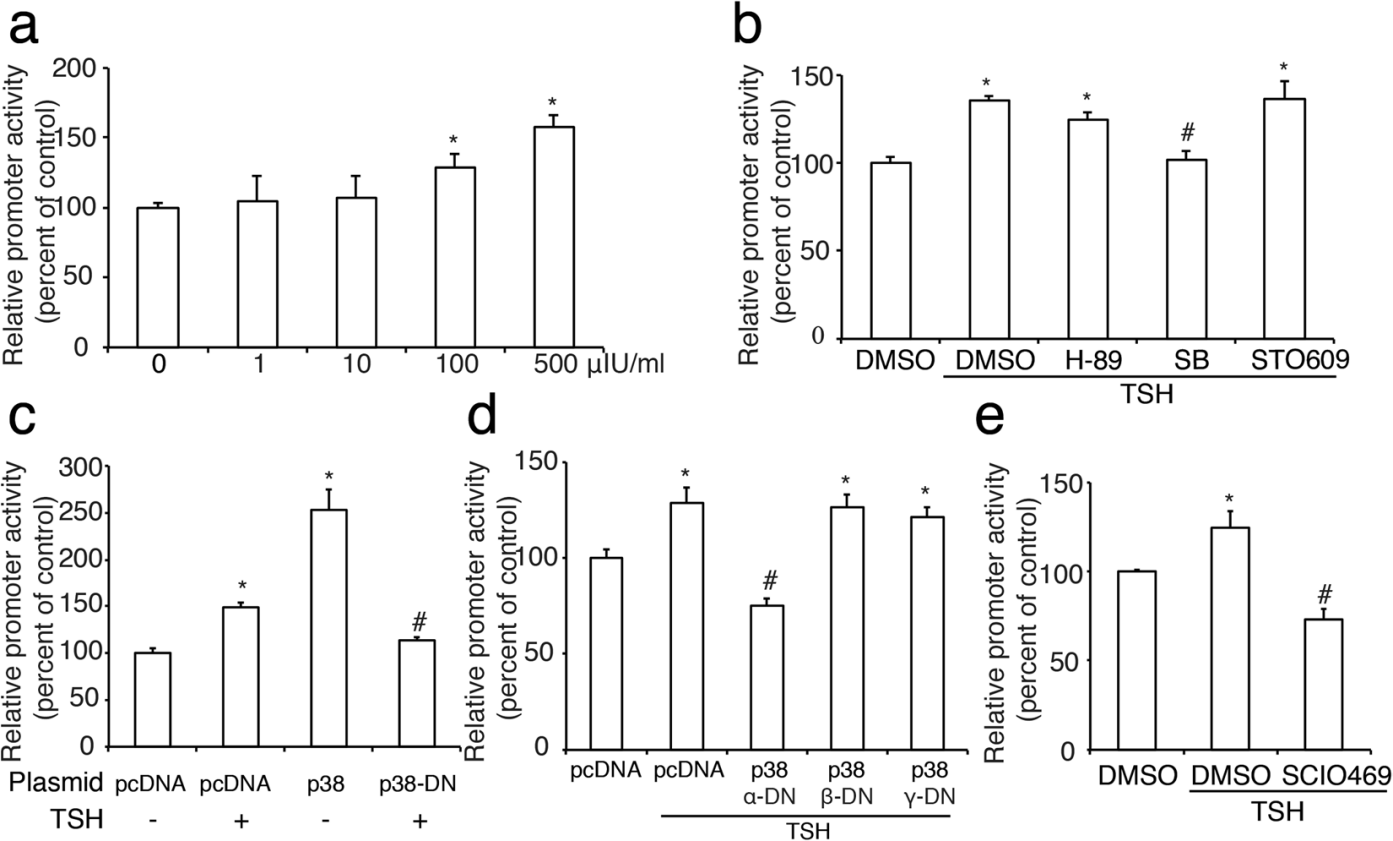
Roles of TSH in the promoter activity of GLUT2 in INS-1 cells. (**a**), TSH increases the promoter activity of GLUT2 in a dose-dependent manner. (**b**) Effects of a PKA inhibitor H-89 (H-89), a p38 MAPK inhibitor SB203580 (SB), or a CaMKK inhibitor STO609 (STO609) on TSH (100 µIU/ml)-induced GLUT2 promoter activity. (**c**) A plasmid containing pcDNA (empty vector), p38 MAPK or p38 MAPK-domain negative (p38 MAPK-DN) DNA was co-transfected into INS-1 cells, along with a plasmid containing the GLUT2 promoter. The cells were then treated with or without TSH at 100 µIU/ml for 24 h. (**d**) A plasmid containing pcDNA (empty vector) or DNA encoding different domain negative subunits (α-DN, β-DN, or γ-DN) of p38 MAPK was each separately co-transfected into INS-1 cells, along with a plasmid containing the GLUT2 promoter. (**e**) Effects of the α subunit of p38 MAPK inhibitor SCIO469 (SCIO469) on TSH (100 µIU/ml)-induced GLUT2 promoter activity. Percentage of promoter activity is relative to control activity level (0, pcDNA or DMSO without TSH), and is shown as the mean ± SEM (n = 3) of separate experiments in the graph. The *denotes a significant difference (P < 0.05) compared to 0, DMSO or pcDNA and the #denotes a significant difference (P < 0.05) compared to pcDNA plus TSH or DMSO plus TSH.

Next, we demonstrated that TSH was able to dose-dependently enhance GLUT2 promoter activity by a luciferase reporter assay (Fig. 3a). To determine the signal transduction pathways through which TSH-induced GLUT2 transcription occurs, we used H-89 (1 μmol/l), SB (SB203580, 1 μmol/l) or STO609 (1 μg/ml) to inhibit the protein kinase A (PKA), p38 mitogen-activated protein kinases (p38MAPK), or Ca^2+^/calmodulin-dependent protein kinase kinase (CaMKK) signaling pathway, respectively. We found that inhibition of the p38MAPK pathway prevented the stimulatory effect of TSH on GLUT2 promoter activity (Fig. 3b), suggesting that p38MAPK may play an important role in this process.

To further investigate this hypothesis, we subsequently cotransfected a plasmid containing p38MAPK or a dominant-negative p38MAPK (p38MAPK-DN) into INS-1 cells along with a luciferase reported plasmid of GLUT2 promoter. We found that overexpression of p38MAPK dramatically stimulated GLUT2 promoter activity, whereas expression of p38MAPK-DN suppressed the stimulatory effect of TSH on GLUT2 promoter activity (Fig. 3c). Next, we evaluated the different roles of p38MAPK α, β and γ subunits in transcription of GLUT2 and found that only a dominant-negative p38MAPK α subunit (p38MAPK α-DN) decreased the effect of TSH (Fig. 3d). Furthermore, a specific inhibitor of the p38MAPK α subunit, SCIO469 (1 μmol/l), produced the same effect as p38MAPK α-DN (Fig. 3e), suggesting that TSH may regulate GLUT2 promoter activity mainly via the p38MAPK α subunit.

**Figure 4:**
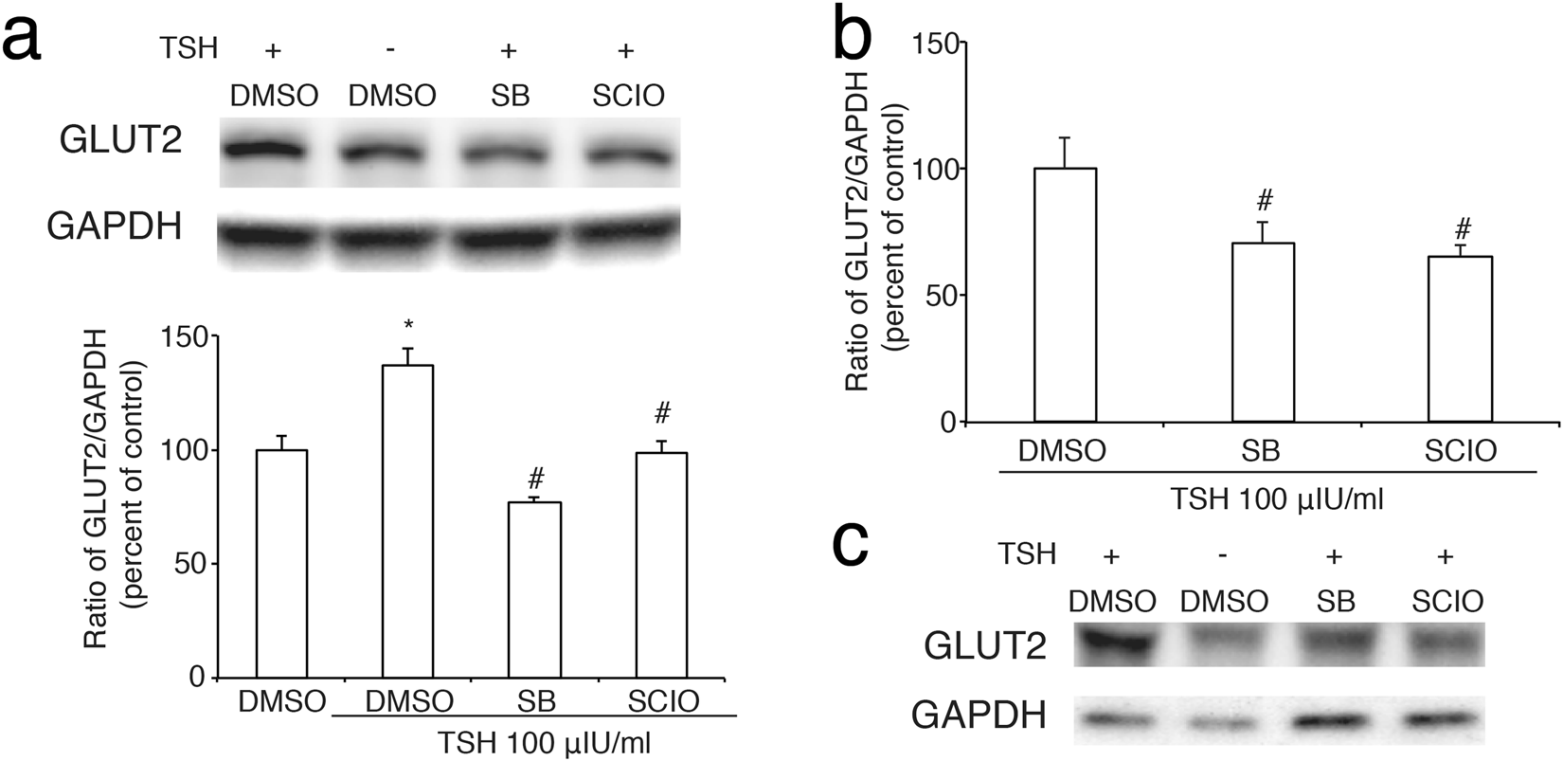
Inhibition of p38-MAPK blocks the effect of TSH on GLUT2 expression in pancreatic *β* cells a and c, protein expression of GLUT2 in INS-1 cells. (**a**) Or pancreatic islets (**c**) which were pre-treated with a p38 MAPK inhibitor SB203580 (SB) or a p38 MAPK inhibitor SCIO469 (SCIO) for 30 min after 6 h starvation and then were incubated with TSH for 24 h. The ratio of GLUT2 to GAPDH is shown as a percentage of the control ratio. (**b**) The effect of SB203580 or SCIO469 on mRNA expression of GLUT2 induced by TSH. GAPDH is used as a control. A graph showing the mean ± SEM (n = 3) of separate experiments for each treatment group is indicated. The *denotes a significant difference (P < 0.05) compared to DMSO; The #denotes a significant difference (P < 0.05) compared to DMSO plus TSH.

Finally, we found inhibition of p38MAPK or its α subunit respectively by SB203580 or SCIO469 significantly blocked the effect of TSH on both protein level and mRNA level of GLUT2 (Fig. 4a and b) in INS-1 cells and it was also demonstrated in primary rat pancreatic islets (Fig. 4c). Taken together, these data pointed out that TSH up-regulates the expression of GLUT2 via p38MAPK, which is mainly via its α subunit.

### TSH induces glucose uptake in INS-1 cells

**Figure 5:**
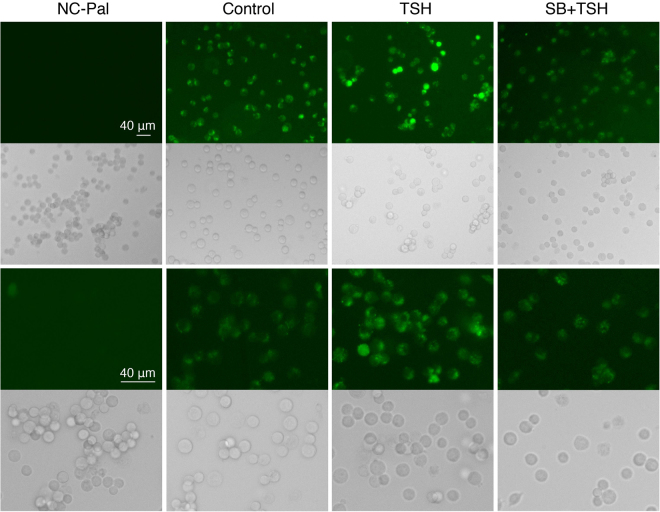
The effect of TSH on glucose uptake in INS-1 cells. After 6 h starvation, INS-1 cells were incubated with DMSO or an inhibitor of p38 MAPK (SB203580) for 30 min, and following treated by TSH at 100 µIU/ml for 24 h. Then the glucose uptake (green) was checked as per the manufacturer’s instruction. NC-Pal served as the negative control and cells treated with DMSO were used as a positive control. The magnification of third and fourth rows are 400 times while the magnification of first and second rows are 200 times. Bar = 40 µm.

Since GLUT2 is a glucose transporter located at the plasma membrane of pancreatic *β* cells, we investigated the effect of TSH on glucose uptake in INS-1 cells. Results showed that glucose uptake was obviously increased upon treatment with TSH, and that this effect disappeared upon inhibition of p38MAPK (Fig. 5), demonstrating that TSH increases glucose uptake via the p38MAPK signaling pathway in pancreatic *β* cells.

### TSH increases the abundance of glucokinase and insulin proteins in rat pancreatic *β* cells

**Figure 6:**
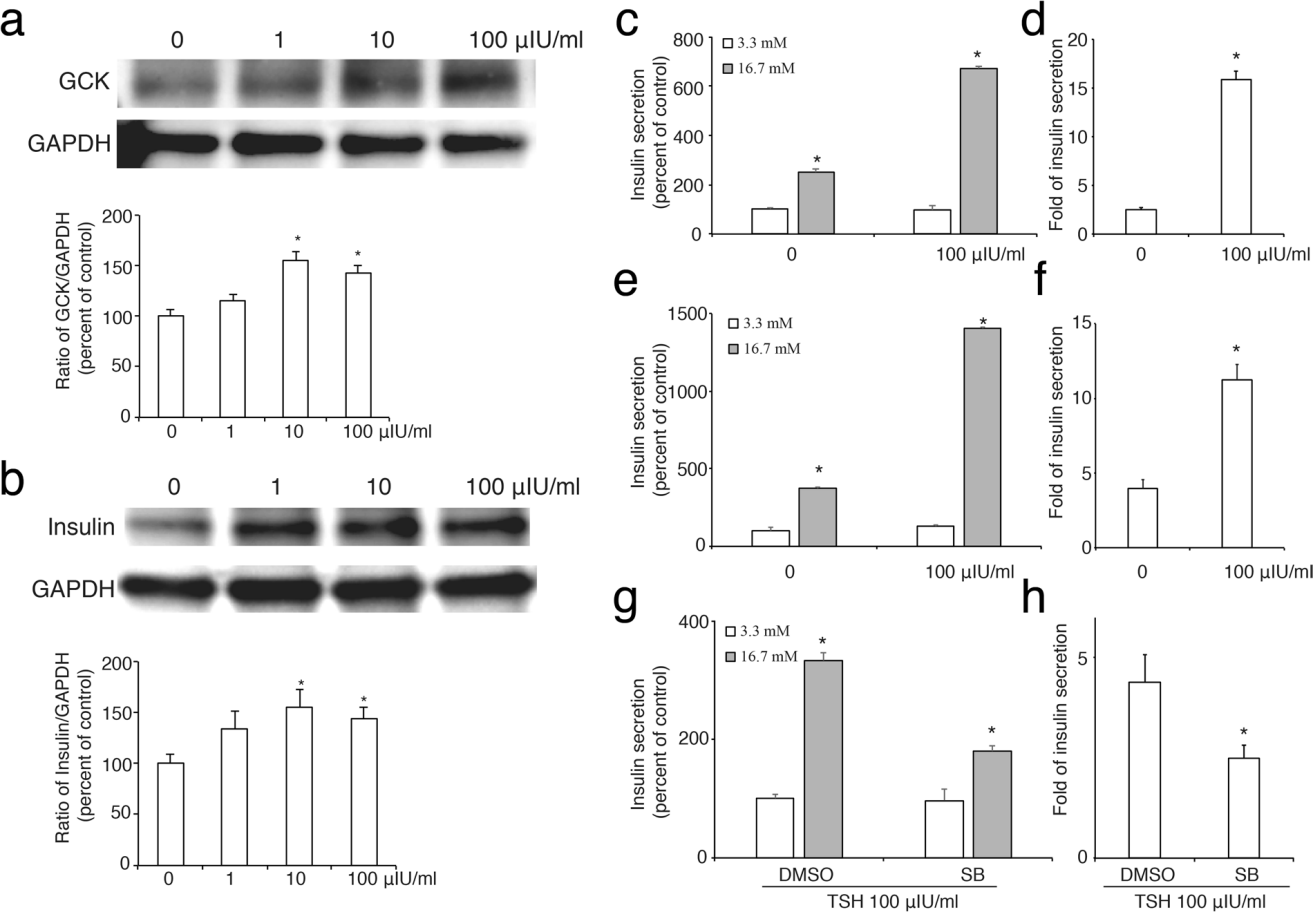
The effect of TSH on the expression of glucokinase and insulin in pancreatic *β* cells. TSH increases the abundance of glucokinase (GK) protein (**a**) and insulin (**b**) in a dose-dependent manner. The ratio of GK or insulin to GAPDH is shown as a percentage of the control ratio. Full-length blots are presented in Supplementary Figure 3. c, e and g, glucose-stimulated insulin secretion (GSIS) in INS-1 cells treated with TSH (**c**) or SB + TSH (**g**) or in pancreatic islets treated with TSH (**e**). d, f and h, fold of insulin secretion from low glucose (3.3 mM) to high glucose (16.7 mM) in INS-1 cells treated with TSH (**d**) or SB + TSH (**f**) or in pancreatic islets treated with TSH (**f**). A graph showing the mean ± SEM (n = 3) of separate experiments for each treatment group is indicated. The *denotes a significant difference (P < 0.05) compared to 0.

It is well established that glucokinase (GK) is a rate-limiting enzyme crucial for the secretion of insulin. The central role of this enzyme in regulating glucose homeostasis provides a strong rationale for studying the regulation of GK expression. As shown in Fig. 6, TSH significantly increased the protein level of both GK (Fig. 6a) and insulin (Fig. 6b) protein synthesis in INS-1 cells.

To further assess the effect of TSH on *β* cells functions, we checked the ability of insulin release in these cells response to high glucose stimulation. As shown in Fig. 6c to e, high glucose (16.7 mM) induced insulin secretion in both INS-1 cells and pancreatic islets from rat and TSH significantly increased the fold of insulin release, indicating that TSH enhances glucose-stimulated insulin secretion (GSIS) in pancreatic *β* cells. However, this effect was dramatically decreased by blockade of p38MAPK with its inhibitor SB203580 (Fig. 6g and h), suggesting that activation of p38MAPK is required in TSH-enhanced GSIS.

## Discussion

In the current study, we built on the results of previously published literature to better elucidate the role of TSH in pancreatic *β* cells. Firstly, we confirmed that TSHR is expressed in both rat pancreatic islets and in INS-1 *β* cell line (Fig. 1). Although previously published literature reports the expression of TSHR in rabbit pancreatic *β* cells^4^, the function of TSH and TSHR in pancreatic *β* cells remains unclear. In this study, we showed that TSH stimulated GLUT2 expression in the INS-1 pancreatic *β* cell line via TSHR, and that TSH also caused increased glucose uptake in INS-1 cells. The stimulatory effect of TSH on GLUT2 promoter activity in the INS-1 cells appears to require the induction of the p38 MAPK cascade.

Glucose transport across the plasma membranes of mammalian cells is achieved via two distinct processes: facilitative transport, mediated by a family of facilitative glucose transporters (GLUT); and sodium-dependent transport, mediated by the Na^+^/glucose cotransporters (SGLT)^17,18^. GLUTs are important for maintaining glucose metabolism homeostasis^19,20^, and are molecular targets of anti-diabetic drugs^21–23^. Thus far, 13 functional facilitative GLUT isoforms have been cloned and characterized, and 12 of these are designated as GLUT1 to GLUT12^24^. GLUT1 through to GLUT5 have been extensively studied, and GLUT2 has been shown to play an important role in mediating glucose-induced insulin secretion from pancreatic *β* cells by catalyzing the uptake of glucose into the cell^25^. Uniquely, only GLUT2-transported sugars are efficient stimulators of the transcription of glucose-sensitive genes^26^, as demonstrated by studies with GLUT2-null mice. These studies showed that the absence of GLUT2 impairs stimulation of glucose-sensitive gene expression, for example, the insulin gene in pancreatic *β* cells, and l-pyruvate kinase in the liver and intestine^27–29^. GLUT2 is generally considered to play a minor role in the glucose-sensing apparatus responsible for the glucose-induced secretion of insulin by pancreatic *β* cells, with glucokinase (GK) being the major player^30^. In this study, we found that stimulation of INS-1 cells by TSH not only increased GLUT2, but also insulin and GK protein expression.

Previously, we reported that the insulin secretagogue hormone glucagon-like peptide-1 (GLP-1) and its long-acting analogue exendin-4 increased the production of not only GLUT2, but also GK and insulin in INS-1 cells^31^. GLP-1 binds to the GLP-1 receptor and activates multiple signaling pathways in the pancreatic *β* cells. These pathways involve a range of protein kinases, including protein kinase A, CaMK, mitogen-activated protein kinases (MAPK, ERK1/2), PI-3K, protein kinase B (*Akt*), and atypical protein kinase C-*ζ*^32^. Moreover, we have identified the role of the CaMKK/CaMKIV signaling pathway in stimulating GLUT2 expression in response to exendin-4^31^. Trafficking of GLUT2 to the membrane is likely to be under the control of PKC βII, as well as the ERK kinase, phosphatidylinositol 3-kinase and p38 MAP kinase intracellular signaling pathways^33^. Of these, activation of the p38MAPK pathway predominates to enhance the expression of GLUT2 gene. The current study shows this to also be true for stimulation of GLUT2 expression by TSH.

p38 mitogen-activated protein kinases (p38 MAPKs) are activated by cytokines, cellular stresses, growth factors, and hormones^34^. p38 MAPKs α, β, and γ subunits are activated by dual phosphorylation of threonine and tyrosine residues within the tripeptide motif TGY by MAP kinase kinases termed MKK3 and MKK6^35,36^. Previous study^37^ investigated the early steps of TSH signal transduction and found that TSH stimulates p38 MAPK phosphorylation and activity in Chinese hamster ovary (CHO) cells stably transfected with the human TSHR. In contrast, TSH does not stimulate the phosphorylation of p38-MAPKs in wild-type CHO cells, indicating that the process is mediated by the TSHR. Interestingly, p38 MAPK appears to be the only MAPK whose phosphorylation is activated by TSH^37^. In this study, we have shown that TSH-dependent activation of GLUT2 is inhibited by a p38 MAPK-specific inhibitor, SB203580, which is reported to be highly specific for p38 MAPK both *in vitro* and *in vivo*, even at concentrations as high as 100 μM^38,39^.

In the thyroid gland, the α isoforms of p38-MAPK is predominantly expressed, whereas expression of the β and γ isoforms is very low^40^. Here, we investigated the roles of three different p38 MAPK isoforms in mediating transcription of GLUT2. Inhibition of the α isoform by both a specific inhibitor, and by its dominant-negative isoform, significantly reduced the stimulatory effect of TSH on GLUT2 promoter activity. This implies an essential role for the p38 MAPK α isoform in this process. However, the way in which it regulates TSH-stimulated induction of GLUT2 expression is unknown. Expression of GLUT2 is regulated by glucose concentration and carbohydrate response element-binding protein (ChREBP) mediates glucose-induced transcription^41,42^. However, the GLUT2 promoter does not appear to contain a ChREBP-binding sequence; rather, it binds sterol regulatory element-binding protein-1C on a sterol-responsive element^43^. Further investigation is required to clarify the mechanisms by which the p38 MAPK cascade mediates transcriptional regulation of the GLUT2 gene.

In Graves’ disease (GD, hyperthyroidism), the main autoantigen (anti-TSHR antibody) activates TSHR without TSH and it is accompanied with high insulin concentration^5,6^. Conversely, in the hypothyroidism state, especially Hashimoto’s disease, the TSH concentration is elevated. Previous studies examined the concentration of insulin in thyroid diseases, and found insulin level to be significantly increased in both hyperthyroid and hypothyroid groups as compared to the euthyroid group^7^. These studies point out that activation of TSHR increases insulin secretion from pancreatic *β* cells and our research also confirmed that glucose-stimulated insulin secretion is significantly induced when TSHR is activated by TSH (Fig. 6). Notably however, insulin resistance, homeostasis model assessment (HOMA) values were also significantly higher in hyperthyroid and hypothyroid groups as compared to the euthyroid group (P < 0.05)^7^. Further study is needed to clarify the detailed mechanism in the effect of TSH on insulin synthesis in pancreatic *β* cells and on the insulin action in its targeted tissue.

In summary, the current study examined the role of the TSH-TSHR signaling in mediating GLUT2 gene expression in the insulin-secreting pancreatic *β* cell line, INS-1. Our results indicate that activation of the p38 MAPK signal transduction pathway by TSH stimulates GLUT2 gene transcription, suggesting that TSH may modify the function of pancreatic *β* cells via TSHR.

## Methods

### Cell culture

The INS-1 cells originated from a rat insulinoma cell line developed and propagated at the Division of Biochimie Cliniqe (courtesy of C. B. Wollheim, Geneva, Switzerland). The present experiments were performed using cell passages 9~35, and the cells being trypsinized every 7 days. These cells were cultured in RPMI1640 media (SIGAMA, Tokyo, Japan) containing 11.2 mM glucose and supplemented with 10% heat-inactivated fetal bovine serum (Dainippon Pharmaceutical Co., Ltd. Tokyo, Japan), 50 μmol/l 2-mercaptoethanol, 100 U/ml penicillin, and 0.1 mg/ml streptomycin in a humidified atmosphere containing 5% CO_2_ at 37 °C. When 80% confluent, the cells were washed twice and incubated with 0.5% fetal bovine serum RPMI 1640 media for 6 h before being stimulated with TSH (SIGMA). The cells were treated with varying doses of TSH for 24 h before harvesting. For the inhibitor treatment, cells were incubated in DMSO or each inhibitor for 30 min before adding TSH.

### Animals

All procedures involving animals were in accordance with Japanese laws and approved by the Animal Care Committee of Kagawa University. Six-week-old Wistar rats were purchased from UNIMEDIA and housed at controlled temperature (25 °C) and lighting (12 h light/dark cycles) in compliance with the Guide for Experimental Animal Research. After 1-week adaptation, animals were sacrificed, and pancreas was extracted for protein extraction, or immediately was fixed by 4% paraformaldehyde (PFA) for immunohistochemistry.

### Pancreatic islets isolation

Isolation of pancreatic islets from rat was performed as described previously^44^. Briefly, the pancreas was digested by collagenase type V at 37 °C for 15 min and then islets were purified and collected by 70 µm cell strainer. Finally, islets (100–200 µm) were picked and placed RPMI1640 media supplemented with 10% heat-inactivated fetal bovine serum, 100 U/ml penicillin, and 0.1 mg/ml streptomycin in 5% CO_2_ incubator at 37 °C. Isolated islets were used for experiments in 7 d.

### Western blot analysis

The 15-µg proteins were separated by a 7.5% sodium dodecyl sulfate (SDS)-polyacrylamide gel and transferred to polyvinylidene difluoride membranes for immunoblotting as described previously^45^. The membranes were blocked overnight at 4 °C and then incubated with 0.1% Tween 20 in phosphate-buffered saline (PBS) (PBS-T) containing the anti-TSH-α antibody (Santa Cruz, 1:200), anti-GLUT2 (Santa Cruz, 1:200), anti-insulin (Santa Cruz, 1:200) or anti-glucokinase (Santa Cruz, 1:200) overnight at 4 °C or anti-GAPDH antibody (Biomol Research, Plymouth Meeting, PA; 1:1000) for 1 h at room temperature. Membranes were then washed three times, 10 min each, with PBS-T and then incubated for 1 h at room temperature with the appropriate horseradish peroxidase-linked secondary antibody (DakoCytomation; 1:2000). Membranes were again washed three times, 10 min each, and antigen-antibody complexes were visualized by ECL (GE Healthcare). Protein bands in Western blot analysis were obtained under Luminescent image analyzer LAS-1000Plus (Fuji Film, Japan).

### Immunohistochemistry

Immunostaining of cells or pancreatic islets was performed as described previously^46,47^. Briefly, cells were fixed in cold (4 °C) 4% paraformaldehyde in 0.1 M phosphate buffer overnight, and pancreas isolated from rats and mice was fixed in formalin and embedded in paraffin. After three-time washing with phosphate-buffered saline (PBS), pH 7.2, fixed cells or tissue sections were incubated with rabbit polyclonal antibody (Ab) TSHR-α subunit (Santa Cruze Biotechnology, Inc.; 1:50) or insulin for 1.5 h at room temperature or overnight at 4 °C. Primary Ab was diluted in PBS/3% BSA/0.03% NaN3/0.2% Triton X-100. After incubated with primary antibodies, cells or sections were rinsed with PBS three times, for 5 min each, prior to secondary antibody application. Then cells were incubated in FITC-conjugated goat anti-rabbit IgG secondary Abs (DakoCytomation; 1:200 diluted in PBS/3% BSA/0.03% NaN3 solution) and rhodamine-conjugated donkey anti-goat immunoglobulins (DakoCytomation; 1:100) for 1 h at room temperature in the dark. Cells or sections were rinsed with PBS three times, for 5 min each and were mounted in 80% glycerol/20% water mix immersion. The processed slides were examined using fluorescence microscopy. Controls treated with nonspecific rabbit IgM or secondary Abs alone showed no staining.

### Polymerase chain reaction (PCR) and real-time PCR

We synthesized cDNA using reverse-transcribed total RNA from INS-1 cells as described previously^48^. INS-1 cDNA was used as the template to get the 597-bp PCR product, TSHR as described in a previous study^16^.

Real-time PCR was performed with a final volume of 10 µl, containing 1 µl of 10 µM each primer, 2 µl of cDNA, 1.6 µl of 25 mM Mg2 + , 1 µl LightCycler-FastStart DNA Master SYBR Green I (Roche) and 3.4 µl sterile PCR-grade H_2_O in tube strips by using CFX96 Touch Real Time PCR Detection Systems (BIO-RAD). The sequences of the forward and reverse primers of GLUT2 are shown as followed: 5′-TTAGCAACTGGGTCTGCAAT-3′ and 5′-GGTGTAGTCCTACACTCATG-3′, respectively. The cycling program consisted of initial denaturation for 10 minutes at 95 °C followed by 45 cycles (35 cycles for ChIP-real time PCR) of 95 °C for 10 seconds, 62 °C for 5 seconds, and 72 °C for 10 seconds with a melting curve of 72-95 °C with a slope of 0.1 C/sec. Each set of PCR reactions included water as a negative control and 5 dilutions of the standard. Known amounts of DNA were then diluted to make the standards, and rat GAPDH was used as the housekeeping standard. The resulting values were analysed by CFX Manager version 1.6 (Bio-Rad), and values were expressed as the relative expression compared to control levels^49^.

### Transfection of INS-1 cells and luciferase reporter gene assay

We used a luciferase reported plasmid (pGLUT2-LUC) containing the promoter region of GLUT2 as previously described^8^. Briefly, 1 μg purified promoter plasmid was transfected into INS-1 using Lipofectamine (Life Technologies, Gaithersburg, MD) with or without co-transfection of p38 MAPK. Transfected cells were maintained in control media containing 100 μIU/ml TSH with or without inhibitors of varying signaling pathway. Transfected cells were harvested, and the promoter activity was measured in an aliquot of the cytoplasmic preparation. 40 μl aliquots were taken for the luciferase assay, which was performed according to the manufacturer’s instructions (ToyoInk, Tokyo, Japan).

### Glucose uptake

For the analysis of glucose uptake, we employed the GluTracker TM glucose uptake assay kit (BioVision, 63671) and carried out the experiment as per the manufacturer’s described.

### Glucose-stimulated insulin secretion (GSIS)

Pancreatic islets or INS-1 cells treated with TSH were starved in Krebs-Ringer bicarbonate (KRB) buffer containing 120 mM NaCl, 5 mM KCl, 1.1 mM MgCl_2_, 2.5 mM CaCl_2_, 25 mM NaHCO_3_ and 0.1% bovine serum albumin (pH 7.4) for 1 h. Following, cells were incubated in KRB buffer containing 3.3 mM glucose for 1 h and then the medium was replaced by KRB buffer supplemented with varying glucose concentration (basal 3.3 mM; stimulatory 16.7 mM) together with other test regents. After 1 h incubation, the supernatant was harvested and used for insulin measurement by ELISA kit (Shibayagi, Japan). All the incubation was performed in 5% CO_2_ incubator at 37 °C.

### Statistical analysis

Statistical comparisons were made by one-way ANOVA and Student’s t test; P < 0.05 was considered significant.

### Data availability

The datasets generated during and analysed during the current study are available from the corresponding author on reasonable request.

## Electronic supplementary material

Supplementary information
