# Supplementary material for: Thyroid stimulating hormone stimulates the expression of glucose transporter 2 via its receptor in pancreatic β cell line, INS-1 cells
Source: Sci Rep. 2018 Jan 31;8:1986. doi: 10.1038/s41598-018-20449-3 (PMC5792451; doi:10.1038/s41598-018-20449-3)

# Thyroid stimulating hormone stimulates the expression of glucose transporter 2 via its receptor in pancreatic *β* cell line, INS-1 cells

Jingya Lyu^1,2^, Hitomi Imachi^1^, Takuo Yoshimoto^1^, Kensaku Fukunaga^1^, Seisuke Sato^1^, Tomohiro Ibata^1^, Toshihiro Kobayashi^1^, Tao Dong^1^, Kazuko Yonezaki^1^, Nao Yamaji^1^, Fumi Kikuchi^1^, Hisakazu Iwama^3^, Ryou Ishikawa^4^, Reiji Haba^4^, Yasunori Sugiyama^5^, Huanxiang Zhang^2*^, Koji Murao^1*^

1. Department of Endocrinology and Metabolism, Faculty of Medicine, Kagawa University, 1750-1, Miki-cho, Kita-gun, Kagawa, 761-0793, Japan
2. Department of Cell Biology, Medical College of Soochow University, Jiangsu Key Laboratory of Stem Cell Research, Ren Ai Road 199, Suzhou Industrial Park, Suzhou 215123, China
3. Life Science Research Center, Kagawa University, 1750-1, Miki-cho, Kita-gun, Kagawa, 761-0793, Japan
4. Department of Diagnostic Pathology, Kagawa University Hospital, 1750-1, Miki-cho, Kita-gun, Kagawa, 761-0793, Japan
5. Department of Life Sciences, Faculty of Agriculture, Kagawa University, 2393, Miki-cho, Kita-gun, Kagawa, 761-0795, Japan.

*** denotes corresponding author.**

*Huanxiang Zhang Ph.D.

Tel: +86-512-6588-0277

E-mail: hzhang@suda.edu.cn

*Koji Murao M.D., Ph.D.

Tel/Fax: +81-878-91-2330

E-mail: [mkoji@med.kagawa-u.ac.jp](mailto:mkoji@med.kagawa-u.ac.jp)

**Supplementary Figure 1 Original Western blot image of Fig 1 a-c.**


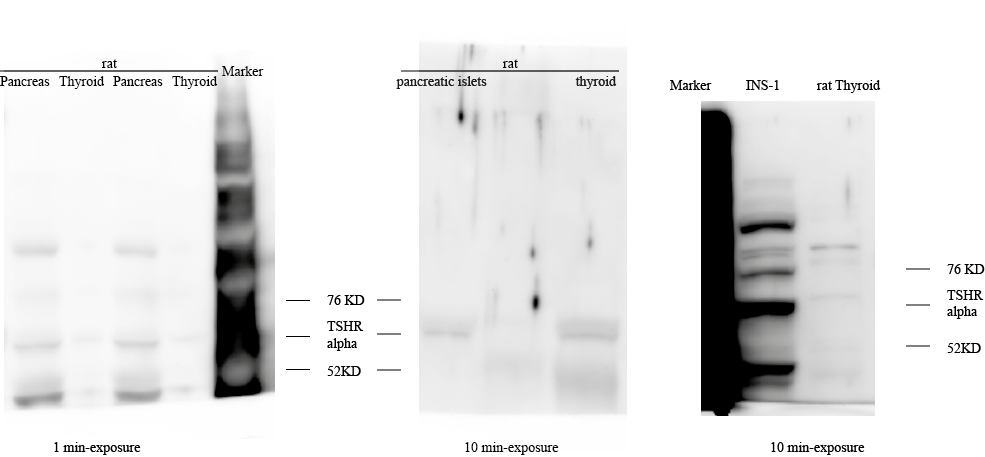


**Supplementary Figure 2 Original Western blot image of Fig 2 a.**


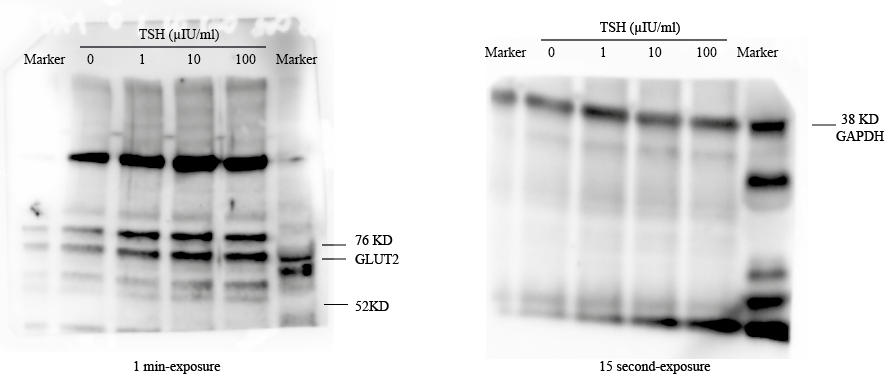


**Supplementary Figure 3 Original Western blot image of Fig 6 a and b.**


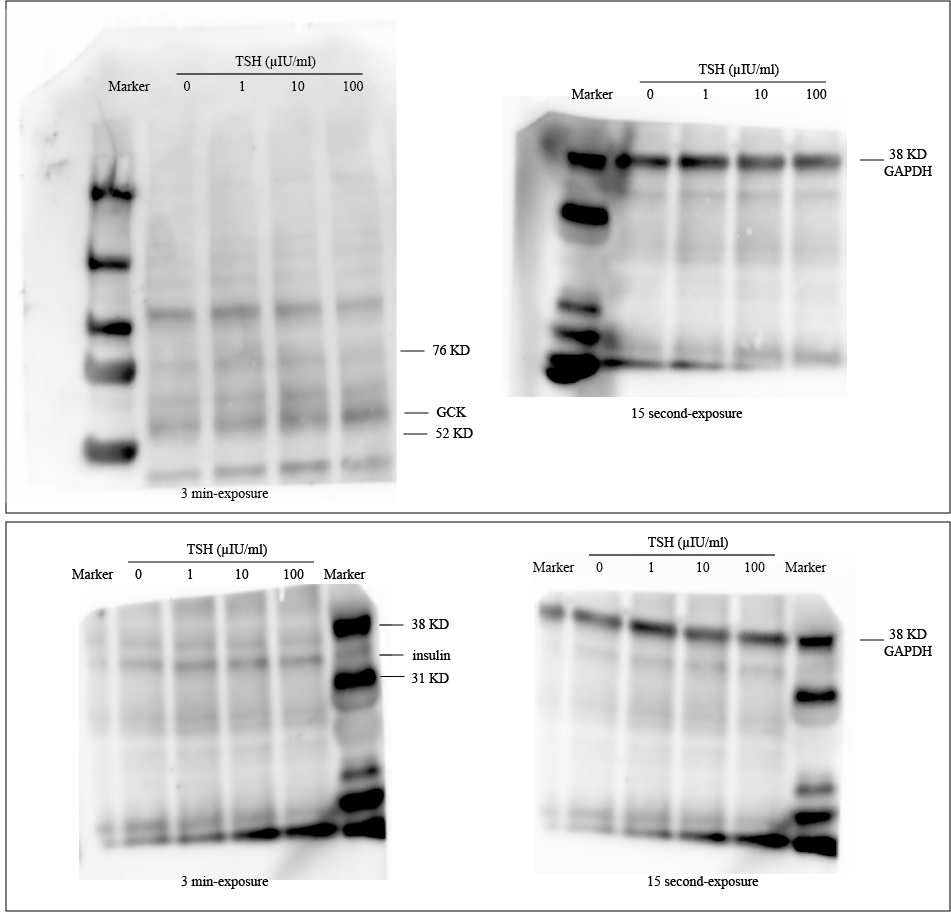

Supplement: Supplementary file 1 — Supplementary information [file 41598_2018_20449_MOESM1_ESM.docx]
